# Dr. Theodor Kocher

**Published:** 1917-09

**Authors:** 


**Dr. Theodor Kocher** of Berne, Switzerland, died Aug. 4. He is said to have performed the first successful operation for goitre, was well known for his work in plastic surgery and received the Nobel Prize for surgery, in 1909. His recent studies had been mainly along the line of a blood preparation to check haemorrhage, probably similar to that already elaborated by Busch and Clowes of Buffalo.

